# Integrating genetic and nongenetic drivers of somatic evolution during carcinogenesis: The biplane model

**DOI:** 10.1111/eva.12973

**Published:** 2020-05-13

**Authors:** Robert A. Gatenby, Stanislav Avdieiev, Kenneth Y. Tsai, Joel S. Brown

**Affiliations:** ^1^ Cancer Biology and Evolution Program Moffitt Cancer Center Tampa FL USA

**Keywords:** biplane model of carcinogenesis, carcinogenesis, fitness function, somatic evolution

## Abstract

The multistep transition from a normal to a malignant cellular phenotype is often termed “somatic evolution” caused by accumulating random mutations. Here, we propose an alternative model in which the initial genetic state of a cancer cell is the result of mutations that occurred throughout the lifetime of the host. However, these mutations are not carcinogenic because normal cells in multicellular organism cannot ordinarily evolve. That is, proliferation and death of normal cells are controlled by local tissue constraints typically governed by nongenomic information dynamics in the cell membrane. As a result, the cells of a multicellular organism have a fitness that is identical to the host, which is then the unit of natural selection. Somatic evolution of a cell can occur only when its fate becomes independent of host constraints. Now, survival, proliferation, and death of individual cells are dependent on Darwinian dynamics. This cellular transition from host‐defined fitness to self‐defined fitness may, consistent with the conventional view of carcinogenesis, result from mutations that render the cell insensitive to host controls. However, an identical state will result when surrounding tissue cannot exert control because of injury, inflammation, aging, or infection. Here, all surviving cells within the site of tissue damage default to self‐defined fitness functions allowing them to evolve so that the mutations accumulated over the lifetime of the host now serve as the genetic heritage of an evolutionary unit of selection. Furthermore, tissue injury generates a new ecology cytokines and growth factors that might promote proliferation in cells with prior receptor mutations. This model integrates genetic and nongenetic dynamics into cancer development and is consistent with both clinical observations and prior experiments that divided carcinogenesis to initiation, promotion, and progression steps.

## 1 INTRODUCTION

What is the definition of cancer? When posed to a wide range of oncologists and cancer biologists, the answers can be remarkably varied. For example, cancer is “a disease of unconstrained growth,” “a disease of the genes,” “uncontrollable cell division,” and “abnormal cells that can invade nearby tissues”. In general, these definitions of cancer describe *properties* of malignant cells or malignant populations. They do not say what cancer *is*. Such definitions may be correct but they are incomplete.

**Figure 1:**
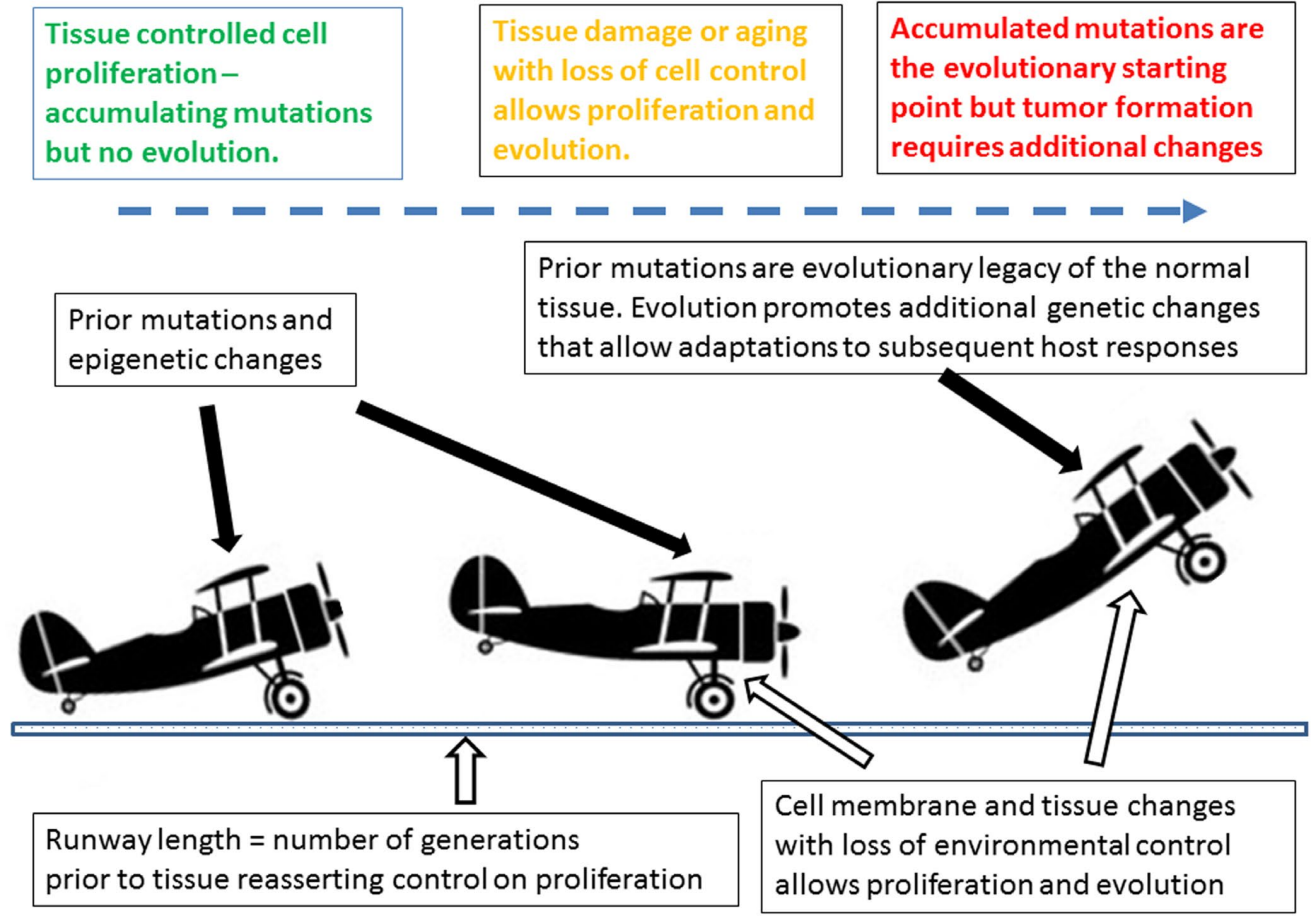
The biplane model of evolutionary dynamics in carcinogenesis. To become airborne, a plane must gain velocity on the runway and have sufficient lift from its wings. In this hybrid model of carcinogenesis, the runway represents the number of cell generations. The initial conditions include a stationary plane—reflecting the absence of evolution in normal cells because their survival, death, and proliferation are determined solely by local tissue constraints. Our model proposes the plane typically can begin rolling down the runway only when it develops a self‐defined fitness when local tissue constraints are lost due to inherent changes in the cell or (probably more often) due to normal tissue disruption caused by injury, inflammation, infection, or aging‐related changes. Once a cell's proliferation is governed only by its phenotypic interactions with local micro‐environmental conditions, it can evolve. As it rolls down the evolutionary runway, the cell's genetic legacy inherited from somatic mutations to the normal cell critically determine the outcome. That is, Darwinian forces can now favor fitness enhancing mutations from those that had accumulated in the cell. If the tissue recovers and attempts to reassert control, natural selection may have already triaged the heritable variation and imbued the cell with the capacity to resist tissue control. At this point, the cell is fully malignant (i.e., airborne)

We propose cancer is a speciation event (Gatenby & Brown, 2017; Gatenby, Cunningham, & Brown, 2014; Vincent, 2010; Vineis, 2003); albeit one that differs substantially from those resulting in the diversity of life that lives around us, rather than in us. Cancer represents a unique evolutionary transition resulting in a new protist (Duesberg & Rasnick, 2000) species derived from host cells (Pienta, Axelrod, Armend, & Brown, 2020). Life, death, location, and function of normal mammalian cells are, under normal conditions, determined solely by instructions from the host tissue. They are necessary for and deeply embedded within the multicellular structure of the host so that each constituent cell has fitness identical to that of the host. To be clear, this evolutionary state does permit competition among, for example, normal stem cells in the bone marrow and skin (Bowling, Lawlor, & Rodriguez, 2019; Ellis et al., 2019; Liu et al., 2019). However, in such contests, we propose the malignant phenotype is not ordinarily achievable because additional tissue controls constrain sustained ecological and evolutionary dynamics (Figure 1).

In contrast, individual cancer cells, while derived from those of the host, have evolutionarily transitioned to a “self‐defined” fitness function (Gatenby & Brown, 2017) in which their survival, proliferation, and death is determined entirely by the Darwinian interactions of their phenotype with critical properties of their local environment. Here, we focus on the cellular and tissue dynamics that can permit a cellular population to transition from one whose fitness is defined by that of the host to one in which each cell has a uniquely defined fitness. Most current models define this transition as a sequence of genetic changes. Others propose entirely nongenetic models in which carcinogenesis is all due to environmental factors (Park, Bissell, & Barcellos‐Hoff, 2000; Sonnenschein & Soto, 2000). Here, we propose a “biplane model” (Figure 1) in which both environmental and genetic factors contribute to cancer evolution through a distinctive sequence of events that can be conceptually centered around the development of a “self‐defined fitness function” (Gatenby & Brown, 2017).

## 2 MATERIALS AND METHODS

### 2.1 Building a conceptual model

A single gram of tumor may contain up to a billion cancer cells, and most cancers are subjected to spatial and temporal fluctuations in blood flow and, therefore, highly variable local environmental conditions (Gillies, Brown, Anderson, & Gatenby, 2018). Thus, each human cancer cell represents the summation of current and prior predictable and stochastic ecological and evolutionary events so that every cancer population represents a species never previously seen on earth. From the perspective of the tree of life, cancer cells across patients do not share a single‐celled common ancestor. This means each cancer is phylogenetically distinct and originated from one or more initially normal metazoan cells (with the notable exceptions of communicable cancers). Within a patient, cancer cells exhibit adaptive radiations into genetically and phenotypically diverse phenotypes and genotypes (also referred to as cancer's Big‐bang (Sottoriva et al., 2015)). Because all cancer cells within the host represent a phylogenetic clade with a common ancestral host cell, their shared heritable phenotypes are termed homologous. Similar heritable phenotypes characteristically seen in cancers from different patients (i.e., the cancer “hallmarks” (Hanahan & Weinberg, 2011)) are termed analogous because their similarity derives from convergent evolution to similar ecological circumstances and not to a shared common ancestor. For instance, wings across all beetle species are homologous, while the wings of a beetle and a bat are analogous (Pienta et al., 2020).

This transition from normal mammalian cell to a single‐celled protist has been described as “atavistic” (Bussey, Cisneros, Lineweaver, & Davies, 2017). There are elements of atavism in the sense that the cancer cells behave as individuals in comparison with normal cells within a multicellular host. Yet, it is misleading to assume that cancer cells have reverted to some evolutionarily primitive state manifestly re‐evolving the states of ancient prokaryotes or eukaryotes. In fact, cancer cells represent forms of progressive evolution in two respects. First, their host tissue environment is unlike anything seen by free‐living single‐celled eukaryotes. Second, in finding heritable variations to fuel evolution by natural selection, the cancer cells have access to the vast information content of the human genome and can, for example, deploy adaptive strategies using genes critical in embryonic development and multicellular organ repair. Such forms of generating and deploying heritable variation are unknown to single‐celled eukaryotes that have not derived from a metazoan; and if they had, this fits the very definition and basis for cancer.

The conventional model of carcinogenesis is “somatic evolution” (Bodmer, 1997) as a cell lineage transitions from normal to cancerous via a stepwise series of intermediate phenotypes. In turn, each step is viewed as resulting from some new mutation. This is classically depicted as a linear process by the model proposed by Fearon and Vogelstein nearly 30 years ago (Fearon & Vogelstein, 1990). More recently, this model of carcinogenesis has been extended to include “branching clonal evolution” as well (Greaves & Maley, 2012). Virtually, all theoretical models of carcinogenesis are built upon the view of evolution as “mutation–selection.” Indeed, the title of the seminal article on cancer as evolution by Cairns (Cairns, 1975) begins with “Mutation selection…” In other words, somatic evolution of a cancer is the result of a mutation that increases fitness within the current environment. Since changes in fitness due to environmental changes are not included in this model, there is an implicit, though perhaps unrecognized, assumption that local selection forces are constant.

### 2.2 Is the genetic model of carcinogenesis complete?

Clearly, cancers are associated with many genetic mutations and targeting key molecular changes (Gatenby & Brown, 2017) can result in tumor regression (although responses are generally temporary). However, here we ask the question: “Is cancer *caused* by genetic mutations and are the hallmarks of cancer cells entirely the result of genetic changes”?

There are, in fact, a number of observations that challenge the gene‐centric model of cancer development and progression (Gatenby, 2017;Yadav, DeGregori, & De, 2016). For example, the average mutational burdens of cancers from different organs can vary by four orders (Martincorena et al., 2015) of magnitude. In contrast, Martincorena et al. (2015) found that only 4–10 driver genes (and sometimes only 1) are likely to be needed for all human cancers. Why would the common hallmarks of cancer require enormous differences in the number of mutated genes necessary to form a malignant phenotype when arising in different organs? Perhaps, as argued by Martincorena et al. (2015) observed mutations beyond the 4–10 critical changes are “excess baggage”—random accumulations of genetic events that have no effect on fitness. In general, however, genetic and phenotypic excess baggage (i.e., the eyes in cave fish) do have subtle evolutionary costs and are, thus, eliminated through the continuous cost/benefit optimization of Darwinian dynamics.

Mutations can be truly neutral and cost‐free if they are silent in the sense of having no consequence for a cell's phenotype. When a cancer cell is far from an evolutionary optimum, such as following initiation, then the likelihood of beneficial mutations is high even as deleterious ones may pose costs. As adaptations accumulate, the cancer cells should evolve to fitness maxima termed an “Evolutionary Stable State (ESS).” At a fitness maximum, any mutation will necessarily produce a less fit phenotype resulting in selection for a progressively small mutation rate. As noted by Pienta et al. (2020), a high mutation rate may facilitate “evolvability” as a trait that allows cancer cell clades to respond to recurring catastrophes in their environment and to track continuous frequent changes in their environment. Yet, as a result of evolutionary triage, following cancer initiation there may be a drop in genetic variability within the cancer cell population relative to that found across the normal cells (Gatenby et al., 2014).

Furthermore, investigations have demonstrated that morphologically normal cells, both adjacent to and distant from a tumor, contain a mutational burden, including in proto‐oncogenes, approaching those present in cancers arising from the same tissue (Deng, Lu, Zlotnikov, Thor, & Smith, 1996; Jamshidi et al., 2017; Martincorena et al., 2015). Similarly, cancer cells transplanted to normal tissue micro‐environments will revert to a normal phenotype (Dolberg & Bissell, 1984). Transfection of a single gene for a Na^+^/H^+^ exchange membrane transporter can cause an immortalized but not malignant fibroblast to become highly invasive (Panet, Marcus, & Atlan, 2000). Cancer is largely a disease that presents in old age, yet most cell divisions and somatic mutations occur early in life (DeGregori, 2013). Finally, across the vertebrate tree of life, neither longevity nor body size positively correlates with cancer incidence (Peto's paradox (Nunney, Maley, Breen, Hochberg, & Schiffman, 2015; Tollis, Boddy, & Maley, 2017)).

In the terminology of Thomas Kuhn (Kuhn, 1996), these observations suggest “anomalies” in the conventional genetic model of cancer initiation, development, and progression. Here, we propose a model of cancer that integrates both genetic and nongenetic mechanisms. Our model is based on classical Darwinian dynamics in which evolution is driven by the interaction of phenotypic properties (i.e., the size and morphology of a Finch's beak) with the properties of a critical selection forces (i.e., the properties of the seed that is the major source of nutrients). We attempt to resolve these anomalies by proposing one obvious and one nonobvious change to the current cancer paradigm. The former simply recognizes that the gene‐centric model of evolution ignores and is not necessary for key components of Darwinian dynamics; particularly those pertaining to the role of changes in environmental selection forces in the transition from a host‐controlled mammalian cell to one that acts as single‐celled species. For the latter, we propose that the genome, while the source of heritable information, is only one component of the complex information dynamics that are necessary to maintain life and drive evolution by natural selection (Gatenby & Frieden, 2017).

### 2.3 Cellular information—is the genetic model complete?

The search for a fuller understanding of the cellular information dynamics arose from the simple observation that the information content of the genome and its products does not equal the information content of the cell. Here, information is quantified in bits as defined by Shannon (Shannon, 1959) although equivalently obtained using mathematical methods described by Kullback‐Leibler (Kullback & Leibler, 1951) and Fisher (Fisher, 1959). For a more comprehensive presentation, see references (Frieden & Frieden, 2004; Frieden & Gatenby, 2011; Gatenby & Frieden, 2007, 2017).

In the 1950s, Morowitz (Morowitz, 1955, 1979), for example, determined that the complexity of the 3‐dimensional structures in a single *Escherichia coli* requires about 2 X 10^11^ bits of information (Gatenby & Frieden, 2005). A similar estimate is obtained using a calorimetric approach (Morowitz, 1955). In contrast, the information storage capacity of the *E. Coli* genome is 10^7^ bits—four orders of magnitude lower (Gatenby & Frieden, 2005)! Of course, genomic information is expanded by translating the information to 100s or 1000s of RNAs and proteins. This information can be estimated using the average molecular composition of *E. coli* and found to be no more than 3.4 × 10^9^ bits (Gatenby & Frieden, 2005)—still 2 orders of magnitude too low*.*

Actually, this exercise simply restates the obvious. There are clearly ordered structures in the cell other than proteins and polynucleotides. Membranes, for example, constitute about 60% of the dry cell mass. In mammalian cells, over 200 different variants of lipid molecules contribute to the membrane and their relative content is precisely controlled. This content varies between different cell types, between different regions of the same cell, and even between the inner and outer layers of the nuclear membrane (Gatenby & Frieden, 2007, 2013). The information content of membranes in a typical mammalian cell has been calculated to be on the order of 5 × 10^10^ bits (Gatenby & Frieden, 2013). While proteins catalyze the formation of lipids, this feed‐forward dynamic is inherently unstable, and clearly, other mechanisms must play a critical role in controlling the lipid distribution.

Transmembrane ion gradients provide somewhat less obvious and under‐appreciated structures for containing information (Gatenby & Frieden, 2017). In mammalian cells, there are asymmetric distributions of Na^+^, Cl^‐^, and K^+^ across cell membranes (manifesting as transmembrane potentials, that is, of −80 to −40mV across the cell membrane) as well as steep transmembrane gradients of Ca^++^ and Mg^++^. Additionally, there is the H^+^ gradient across the mitochondrial membrane. There are even ion gradients, recently reported, across the nuclear membrane. Such highly improbable transmembrane ion distributions (compared to the expected equal concentrations in the intra‐ and extracellular fluid) can be calculated using Shannon entropy. In *E. Coli*, which is much smaller than a typical mammalian cell and lacks mitochondria and a nucleus, the information content of the transmembrane ion gradients turns out to be just over 10^11^ bits (Gatenby & Frieden, 2005)—vastly larger than the information in the genome.

These data have led to the proposal that there are two distinct but integrated information systems in each cell. This includes the well‐recognized heritable but fixed information in the genome. However, a critical function of the genome is to produce macromolecules that act like “Maxwell's demon” by using energy and information to produce large nonequilibrium structures (i.e., the transmembrane ion gradients) that (similar to eyes, ears, etc.) gain spatial and temporal information from the environment allowing the cell to adapt to a wide range of conditions throughout its lifetime. In normal tissue, these information dynamics allow cells to form multicellular structures with complex but robust spatial and functional organization (Gatenby & Frieden, 2007).

Our fundamental hypothesis is that both information dynamics contribute to multicellular dynamics and so both must undergo substantial changes for transition of a single cell from a normal component of the tissue society to one that is, in effect, a single cell protist.

## 3 RESULTS

### 3.1 Synthesizing genetic and nongenetic information dynamics—An evolutionary model of carcinogenesis

How does a normal mammalian cell develop a self‐defined fitness function? Fundamentally, this depends on the local tissue environment that governs control of cellular proliferation and death. In multicellular organisms, maintaining the structure and function that allows survival and reproduction requires tight control of its constituent cells. They must be controlled by host instructions so that their position, differentiated state, and activities serve the fitness of the multicellular organism. A normal cell's survival and proliferation is subordinate to the whole. In a sense, the collective behavior of all cells is a team optimum serving the whole organism. Because their proliferation and death (in the absence of infection or trauma) are controlled entirely by the host instructions, normal mammalian cells do not have the capacity for much evolution. That is, while competition among normal stem cells is observed, the host retains sufficient control to constrain Darwinian events beyond those that optimize local tissue function. In contrast, a cancer cell has broken from this team optimum and lost all connection to the host instructions. In this setting, survival, death, and proliferation are entirely dependent on the interactions of its phenotypic properties with selection forces in the local tissue micro‐environment. These strategies may include exploiting host‐generated factors (e.g., estrogen in ER‐positive breast cancers) but here they act as resources and not explicit instructions. Thus, for example, if estrogen concentrations decline, the cancer cells will evolve alternative methods to maintain growth in the absence of ligand binding receptors (e.g., constitutive upregulation of the estrogen pathway). In contrast, the normal cells will simply stop proliferating and/or die as evidenced by changes in the breast parenchyma during the menstrual cycle.

Thus, the dynamic that fundamentally determines carcinogenesis is loss of organismal control of an individual cell or group of cells. Note that a “normal” mammalian cell may accumulate many somatic mutations (including those that would ordinarily be oncogenic) without becoming a cancer provided their proliferation remains fundamentally regulated by the collective that is the whole organism. In the conventional model of carcinogenesis, this loss of control occurs through a series of mutations that progressively renders the cell unable to receive tissue signals. This is certainly a reasonable hypothesis. But, as noted above, variations in the number of mutations in cells from different tissues of origin and the presence of a similar mutational load in both cancer and adjacent normal cells do not appear to be consistent with this model.

There is an alternative somatic evolutionary arc in which the cell initially retains the capacity to respond to host signals. However, carcinogenesis begins when tissue signals are lost following damage to the tissue itself. Note this signal loss does not need to be genetic but could occur within the non‐genetic information processing at the cell membrane. Thus, the first steps toward cancer may result from changes in the local tissue and not the cell. That is, the cell becomes free to evolve not because accumulating mutations have rendered it unresponsive to local tissue control but rather because the disordered local tissue can no longer exert that control. To be clear, once the transformed cell begins to evolve, it is subject to selection pressures in the environment. If the local tissue is injured or infected, the environment may include cytokines which could promote proliferation of local cells that happened to have acquired a cytokine receptor mutation in the past. That is, a genetic change that caused no changes when the cell was controlled by the local tissue can promote fitness when the tissue is damaged.

Thus, the phenotypic properties encoded in a cell's genome (including accumulated mutations throughout the prior lifetime of the host) will determine fitness so that the genetic changes from this baseline state will represent a history of the adaptive strategies required during the evolutionary arc of each cancer cell. Note this model predicts the number of mutations in cancers from different organs reflect the typical accumulation for that organ rather than the necessary number of mutations to form a cancer. Thus, for example, cancers arising in the skin have larger number of mutations than cancers arising in the kidney because normal skin cells, exposed to UV light, simply accumulate more mutations over a lifetime than do normal kidney cells. because normal skin cells are exposed to mutagens some 

In this model, we assume that the perturbation to local tissue is most commonly transient. That is, following successful repair of tissue following trauma, inflammation, and infection, the reconstituted tissue re‐asserts control. In theory, we expect this will often cause cells that had a self‐defined fitness function to respond, become suppressed and revert to their ancestral role in support of the functioning tissue. However, because the local cell population can evolve, we expect that, under some circumstances, one or more cells will accumulate heritable changes that allow them to maintain an independent state. Thus, populations of the former cell type will regress while those with the latter will continue to proliferate and become a speciation event to a new single‐celled organism. Furthermore, tissue subject to repeated injury or inflammation may over time accumulate cells that are increasingly likely to become permanently independent (i.e., cancer cells) upon a subsequent perturbation.

Is there evidence for this hypothesis? In fact, this mechanism of carcinogenesis has been hiding in plain sight through decades of experiments that investigate “initiation, promotion, and progression” in cancer development. Briefly, the experiment (Hennings et al., 1993) typically begins with the application of a mutagen that increases mutations in a local population (usually but not always on the skin). Although mutations are documented, the cells remain phenotypically normal with no increase in proliferation. If initiation is followed by multiple applications of an irritant that causes inflammation, nonmalignant tumors (papillomas) develop. Nearly all these tumors then regress, and spontaneous conversion to cancer is rare. However, the rate of conversion can be increased by the application of genotoxic agents.

In the context of our proposed model, the inflammation of the skin, by disrupting the information dynamics in the cell membrane, causes loss of control in local cells. That is, normal proliferative (stem) cells at the site of inflammation or injury no longer receive control signals from local tissue. As a result, their survival and proliferation become dependent on their phenotypic properties and their interactions with local micro‐environmental conditions. In evolutionary terms, these cells now have a self‐defined fitness function which allows them to proliferate repeatedly, compete with other cell clades, and experience cell turnover, and thus, they can begin to evolve. Thus, there are two general pathways to this state. First, the individual cell could become uncontrollable by local tissue signals if the reception mechanisms are lost either due to mutations or disruption of the membranes. Second, a cell could become independent because of damage to the local tissues which results in absent or uncoordinated signaling by the tissue. Although these cells may be identical to nearby “normal cells” (including similar mutational burdens), they can now evolve simply because their survival and proliferation are no longer controlled by the damaged local tissue.

The number of cells that can proliferate is expanded by the genetic changes caused by the preceding initiation step. This produces the cell growth observed as a papilloma. When the tissue damage resolves, normal tissue controls result in regression of nearly all cells with resolution of the papilloma. However, some cells in the papilloma have, likely by chance, acquired genetic or epigenetic changes that produce a phenotype that is no longer controllable by the local tissue. The addition of genotoxic stress increases the probability that the resistant phenotype will develop allowing the cells to maintain a self‐defined fitness function. The clade with a cell‐defined fitness function has now speciated. Thus, the jump to cancer requires speciation but must be preceded by opportunities for normal cells to have unusually long runs of cell proliferation and turnover. This allows them to have self‐defined fitness functions and become subject to natural selection.

If the development of a self‐defined fitness function is not genetic, what is the mechanism? We see it as emerging from unusually long runs of cell division and turnover. Such runs can happen when there is tissue repair that can and must be filled, and when there is a temporarily and spatially localized cessation of external tissue control on a clade's proliferation. Both involve information dynamics on the cells’ membrane and within the surrounding extracellular matrix. The information dynamics within the multicellular tissue become altered during periods of wounding and inflammation.

Several empirical observations support the critical role of information dynamics on the cell's membrane for multicellular tissue repair and homeostasis. For example, the transmembrane potential is directly related to proliferation and to cancer formation (Yang & Brackenbury, 2013). More recent studies find extensive spatial variation in the potential throughout the surface area of the cell membrane (“like a soccer ball”) (Morokuma et al., 2008). Furthermore, changes in the cell membrane potential are integrally related to formation and progression of cancer populations (Berzingi, Newman, & Yu, 2016; Yang & Brackenbury, 2013) and membrane depolarization has been linked to activation of ERK (Waheed et al., 2010)—a well‐known mediator of oncogenic signaling in cancer (Dhillon, Hagan, & Rath, 2007).

### 3.2 Runway and lift hypothesis for carcinogenesis

Metaphorically, our model for carcinogenesis can be likened to the successful takeoff of an airplane (Figure 1). The runway provides space to gain speed, and the airplane's lift determines the critical speed at which takeoff happens. Natural selection requires creative destruction, namely cell turnover via births and deaths. A million normal cells where each turns over five generations have little chance of developing cancer. But a single cell that turns over 5 million times will almost certainly become cancerous. In our model, having an unusually long run of cell divisions and turnover represents the runway permitting a self‐defined fitness function. Inflammation or chronic wounding provides some progenitor cells with long runways. Mutations, epigenetic changes, and alterations to the cell membrane that renders the cell independent of normal tissue control represent lift.

If one pushes the metaphor, the airplane can be thought of as a biplane with one wing representing the classic model of carcinogenesis via driver mutations. These driver mutations may already be present or accumulate as the cell experiences its run of cell divisions. But, the cell's chance at cancer stops, perhaps permanently, once the run of cell divisions stops and normal tissue control resumes—the runway has ended.

A second process may provide lift and the second wing of the biplane; and this second source of lift may be as, or perhaps even more important than the accumulation of new driver mutations, the each will facilitate the other. We hypothesize that the second source of lift comes from nongenetic mechanisms that temporarily or permanently alter the cell membrane or other aspects of signaling pathways to maintain cell turnover, even as the tissue attempts to reassert control over proliferation.

In this metaphor, we see why UV radiation and smoking become potent promoters of skin cancers and lung cancer, respectively. Both induce wounding and inflammation, thus increasing the length of the runway. Both also act as mutagens and cell metabolic disrupters adding lift to both wings of the biplane.

The cell lineage that gains speed via cell turnover takes off and becomes a malignant cancer cell by exhibiting four of the hallmarks of cancer (Hanahan & Weinberg, 2011): resisting cell death, replicative immortality, evading growth suppressors, and sustaining proliferative signaling. Many properties of tissue regulation and wound healing are anticancer adaptations that shorten the runway and reduce lift. P53 and other genes that either promote DNA repair (Gao et al., 2000) or initiate apoptosis (Fridman & Lowe, 2003) on damaged cells reduce lift, and these are well appreciated. Less well appreciated are mechanisms to keep the runway short. These include asymmetric cell division with concomitant cell differentiation into nonproliferative states, telomere shortening, and the need to adhere to a basal membrane for sustained proliferation.

This concept may also explain some of the unusual features of familial cancer predisposition syndromes. Often, these have remarkable tissue specificity and even limited time windows of emergence. One example is retinoblastoma, an exclusively pediatric tumor of the developing retina largely driven by mutations or deletions in the *RB1* gene. While complete loss of function of this one gene appears sufficient to provide the necessary lift and drive tumor formation, the runway is only open for a short time in childhood when there are actively dividing retinal progenitor cells.

Virus‐associated cancers reflect a broad range of potential activities. High‐risk alpha human papillomaviruses and Merkel cell polyomavirus, for example, co‐opt and modify aspects of the cell's machinery so as to forestall cell differentiation and promote longer runs of cell divisions. As nonlytic viruses, this makes sense from the virus's perspective as it permits greater numbers of virions via intrinsically sustained cell divisions of infected cells (Orlando, Gatenby, Giuliano, & Brown, 2012). This lengthens the runway as well as adding lift to the nongenetic wing of the biplane; facilitating HPV induced cervical cancer and Merkel cell carcinoma. In contrast, chronic viral hepatitis predisposes to hepatocellular carcinoma through both genetic means (viral genes driving aberrant transcription) as well as inciting inflammatory responses (for example resulting in cirrhosis). In this case, the path to cancer involves elevated mutations rates (genetic lift) and increased runway length through reduced extrinsic tissue control over proliferation.

This lift and runway model invites an expanded view of carcinogenesis that goes beyond simply focusing on the slow, lifetime accumulation of mutations (one wing of the biplane). We see critical roles for (a) environmental perturbations (wound healing, aging, and inflammation) giving rise to long runs of cell division with less and varied amounts of tissue control (lengthening the runway) and (b) changes in cell membrane functioning due to intrinsic cellular properties or alterations to a cell's properties due to the absence of extrinsic tissue control mechanisms (second wing of the biplane). Together, these can allow a cell clade to transition into a self‐defined fitness function that permits somatic evolution and eventual speciation into a new single‐celled pathogen. All three aspects of the biplane model (long runs of cell division, genetic mutations, and alterations to information dynamics on the cell membrane) may be necessary for oncogenesis; and none by themselves may be sufficient. Each of these may be exacerbated by genetic predispositions and alterations to the immune system or tissue control mechanisms due to illness or age.

## 4 CONCLUSION

The classic model of carcinogenesis as a sequence of random mutations to critical genes corresponding to progressively more malignant phenotypes is often termed “somatic evolution” and is based on the assumption that evolution proceeds through a “mutation–selection” sequence. However, this represents a narrow view of Darwinian dynamics, which are fundamentally governed by the interaction of local environmental properties acting as selection pressures with the phenotypic properties of the evolving organisms. That is, in evolution by natural selection, the genome is a mechanism of inheritance so that accumulating changes serve more as a history of the organism's experiences rather than its cause.

We present an alternative model in which carcinogenesis is viewed as a transition of cellular fitness functions. For a normal mammalian cell, fitness is identical to that of the multicellular organisms because it is controlled by local tissue instructions. In contrast, a cancer cell has a “self‐defined” fitness function because its survival, death, and proliferation are determined by the interactions of its phenotypic properties with local environmental selection pressures. Most importantly, this transition allows the transformed cell to become a new species that can evolve and adapt to conditions within the host ultimately allowing it to proliferate independently. The cancer cell goes from being part of the whole organism's fitness function to becoming its own unit of selection. Loss of tissue control can be integrated with the gene‐centric view of carcinogenesis by noting how the accumulation of mutations may render the cell unable to receive or process extracellular tissue signals.

Here, we propose an alternative model in which a cell can acquire a self‐defined fitness function due to loss of normal tissue controls caused by local tissue disruptions such as inflammation, infection, or injury. In this model, genetic mutations can accumulate in normal cells, based on random events and exposure of the tissue to mutagens (i.e., skin and UV radiation), but these have no effect on the cellular phenotype provided the tissue controls persist. However, when normal tissue controls are lost or suspended, the cell can use its normal and mutant genetic heritage to adapt to local tissue conditions and, thus, evolve. What follows is a race for control. The local tissue will typically be repaired so that it is able to reestablish control (the runway has finite length). This adds new selection forces to the environment. Cells that do not possess adaptive strategies will revert because of tissue control and remain “normal” (an aborted takeoff). On the other hand, some progenitor cell(s) will possess prior mutations (lift from upper wing), and it will acquire additional mutations, epigenetic switches, and changes to membrane signal processing (lower wing of the biplane) during its long run of proliferations within the damaged tissue. Hence, such a cell lineage may have acquired ways to counter or ignore the re‐emergence of external tissue controls. This clade will continue to proliferate have sufficient lift to takeoff and become a single‐celled species capable of subsequent evolution as cancer.

We note how this model is consistent with accumulating clinical data that the mutational burden of normal cells adjacent to a cancer can approach that of the malignant cells within the tumor. Thus, the observed differences in mutational burdens of cancers from different organs (up to 10,000 fold) represent the underlying mutation rates in those tissues as a combined readout of exposure to mutagenic insults, repair, and proliferation, rather than some intrinsic likelihood that cancer cells will arise in that tissue. Finally, we demonstrate how the dynamics observed in classical initiation, promotion, and progression experiments are consistent with our proposed model (Hennings et al., 1993;Vincent & Gatenby, 2008).

We feel our model is relevant to clinical cancer prevention strategies. It provides a mechanistic explanation for why and how interventions that reduce inflammation and infection decrease the probability of cancer development ( Rayburn, Ezell, & Zhang, 2009). It breaks cancer initiation and progression into three phases where the first represents wounding or tissue damage that creates long runs of cell division, the second sees the cancer cells as gaining a self‐defined fitness function that sees changes in cell membrane information processing, and third is the emergence of a new single‐celled pathogen that can now be subject to natural selection.

Toward forestalling and stopping cancer at its onset we can imagine a sequence of therapeutic interventions aimed at each phase. For example, in phase 1, when the inflammation and wounding associated with tissue damage creates unusually long runs of cell division, an anti‐inflammatory may prevent unusually long runs of cell divisions. In phase 2, a targeted chemotherapy may be most effective as it will control those clones that have emerged with mutations or changes to signaling pathways that slow cell differentiation and speed proliferation. In phase 3, an immunotherapy may be most effective at treating any emerging cancerous lesions as these cells will begin to evolve adaptive traits that present antigens susceptible to immune attack.

## CONFLICT OF INTEREST

The authors declare no conflicts of interest.

## Data Availability

No new data are included. All presented data are publicly available in the referenced articles.
